# Channel Properties of Na_x_ Expressed in Neurons

**DOI:** 10.1371/journal.pone.0126109

**Published:** 2015-05-11

**Authors:** Masahito Matsumoto, Takeshi Y. Hiyama, Kazuya Kuboyama, Ryoko Suzuki, Akihiro Fujikawa, Masaharu Noda

**Affiliations:** 1 Division of Molecular Neurobiology, National Institute for Basic Biology, Okazaki, Japan; 2 School of Life Science, SOKENDAI (The Graduate University for Advanced Studies), Okazaki, Japan; Universidad de La Laguna, SPAIN

## Abstract

Na_x_ is a sodium-concentration ([Na^+^])-sensitive Na channel with a gating threshold of ~150 mM for extracellular [Na^+^] ([Na^+^]_o_) *in vitro*. We previously reported that Na_x_ was preferentially expressed in the glial cells of sensory circumventricular organs including the subfornical organ, and was involved in [Na^+^] sensing for the control of salt-intake behavior. Although Na_x_ was also suggested to be expressed in the neurons of some brain regions including the amygdala and cerebral cortex, the channel properties of Na_x_ have not yet been adequately characterized in neurons. We herein verified that Na_x_ was expressed in neurons in the lateral amygdala of mice using an antibody that was newly generated against mouse Na_x_. To investigate the channel properties of Na_x_ expressed in neurons, we established an inducible cell line of Na_x_ using the mouse neuroblastoma cell line, Neuro-2a, which is endogenously devoid of the expression of Na_x_. Functional analyses of this cell line revealed that the [Na^+^]-sensitivity of Na_x_ in neuronal cells was similar to that expressed in glial cells. The cation selectivity sequence of the Na_x_ channel in cations was revealed to be Na^+^ ≈ Li^+^ > Rb^+^ > Cs^+^ for the first time. Furthermore, we demonstrated that Na_x_ bound to postsynaptic density protein 95 (PSD95) through its PSD95/Disc-large/ZO-1 (PDZ)-binding motif at the C-terminus in neurons. The interaction between Na_x_ and PSD95 may be involved in promoting the surface expression of Na_x_ channels because the depletion of endogenous PSD95 resulted in a decrease in Na_x_ at the plasma membrane. These results indicated, for the first time, that Na_x_ functions as a [Na^+^]-sensitive Na channel in neurons as well as in glial cells.

## Introduction

Na_x_ is a sodium (Na) channel that was originally cloned independently from rat astrocytes [1], the human heart [2], a mouse atrial tumor cell line [3], and rat dorsal root ganglia [4]. Na_x_ is a member of the voltage-gated Na channel family, but markedly differs in key regions for voltage sensing and inactivation [5–8]. The generation of *Na*_*x*_-knockout (*Na*_*x*_-KO) mice by insertion of the *lacZ* reporter gene in-frame allowed us to visualize the distribution of *Na*_*x*_-gene expression [9]. The dense signals of *lacZ* were shown to be limited to glial cells in some brain regions, including the subfornical organs (SFO) and organum vasculosum of the lamina terminalis (OVLT), and median eminence in the central nervous system (CNS) [9, 10]. Futhermore, the relatively weak expression of *lacZ* was observed in the neurons of some brain regions, including the cerebral cortex in layer IV of the lateral area and the amygdala [9]. In the peripheral nervous system (PNS), *Na*_*x*_ is expressed in non-myelinating Schwann cells and neurons in the dorsal root ganglia (DRG) [9, 11].

Functional analyses have revealed that Na_x_ is a Na^+^ concentration ([Na^+^])-sensitive, but not a voltage-sensitive Na channel with a threshold of ~150 mM for extracellular [Na^+^] ([Na^+^]_o_) *in vitro* [12]. *Na*_*x*_-KO mice did not stop ingesting salt even when dehydrated, while wild-type mice avoided salt. This defect was recovered by the site-directed transfer of the *Na*_*x*_ gene into the SFO, suggesting that glial cells in the SFO are the primary site for [Na^+^] sensing in order to control salt-intake behavior [13]. These findings indicated that Na_x_ is a sodium sensor that detects increases in [Na^+^] in the blood and cerebrospinal fluid (CSF). As subsequent study revealed that glial cells expressing Na_x_ in the SFO used lactate as the gliotransmitter to transmit information on [Na^+^] increases in body fluids from glial cells to GABAergic neurons in the SFO [14].

Na_x_ has a PSD95/Disc-large/ZO-1 (PDZ)-binding domain at the carboxyl (C)-terminus [15]; the C-terminal sequence of Na_x_ (–Q–T–Q–I for the rat and mouse, and –Q–S–Q–I for humans) fits a ‘non-canonical’ PDZ-binding motif (–X–S/T–X–I/A). PDZ-binding domains are protein-protein interaction modules that bind specifically to their target PDZ proteins. We screened for potential interacting proteins with the PDZ-binding motif at the C-terminus of Na_x_. Several PDZ proteins were identified by the PDZ-array overlay assay using the glutathione S-transferase (GST)-fused protein with the C-terminal region of Na_x_ [15]. Of these proteins, we found that SAP97, a member of the membrane-associated guanylate kinase (MAGUK) family, was co-expressed with Na_x_ in glial cells in the SFO [15]. Further analyses using C6 glioblastoma cells revealed that SAP97 contributed to the stabilization of Na_x_ at the plasma membrane [15].

In the present study, we demonstrated that Na_x_ was expressed in some neurons in the amygdala. We established a cell line from mouse neuroblastoma Neuro-2a cells that exogenously expressed *Na*_*x*_ when induced with a drug. Using this cell line, we demonstrated that the [Na^+^] sensitivity of Na_x_ in Neuro-2a cells was similar to that in C6 glioma cells. We also found that Na_x_ bound to PSD95 through its PDZ-binding motif at the C-terminus. The knockdown of endogenous PSD95 led to a reduction in the cell-surface expression of Na_x_, suggesting that PSD95 in neurons contribute to the stabilization of Na_x_ at the plasma membrane.

## Materials and Methods

### Ethics statement

All experimental protocols with animals were approved by The Institutional Animal Care and Use Committee of National Institutes of Natural Sciences, Japan; approval numbers are 12A051, 13A082, and 14A149. All surgeries were performed under sodium pentobarbital anesthesia, and all efforts were made to minimize suffering.

### Experimental animals

Adult rats (Sprague-Dawley, CLEA Japan), wild-type mice (C57BL/6J, CLEA Japan), Thy1-yellow fluorescent protein (YFP) transgenic mice [B6.Cg-Tg (thy1-YFP)16Jrs/J, Jackson Laboratory], and homozygous *Na*_*x*_-KO mice were used for experiments.

### Primary culture

The lateral amygdala was dissected from newborn mice (5–10 days), and dissociated cells with papain at 37°C for 1 h were collected by centrifugation (800 g for 5 min). They were then plated on a glass-bottomed dish coated with 100 μg/ml poly-D-lysine and cultured with neurobasal medium containing B-27 and GlutaMAX I (Life Technologies) in a humidified incubator at 37°C with 5% CO_2_ for 3 days.

### Cell lines

Mouse neuroblastoma Neuro-2a cells (CCL-131, ATCC), C6 rat glioma cells (CCL-107, ATCC), and HEK293T cells (human embryonic kidney cells) (CRL-3216, ATCC) were obtained from ATCC. Cells were grown and maintained in Dulbecco's Modified Eagle Medium (DMEM) supplemented with 10% fetal calf serum (FCS). Regarding the differentiation of Neuro-2a cells, the medium was replaced with DMEM containing 1 mM dibutyryl cyclic adenosine monophosphate (dbcAMP) or 20 μM retinoic acid and cultured for 48 h.

C6M16 cells, a C6 cell line in which the expression of mouse Na_x_ is inducible under the control of the tetracycline-responsive element (TRE), was described previously [14]. To induce the expression of Na_x_, a Tet-off adenoviral vector (Clontech) was added to the medium.

The C6Mf4 and N2a-Mf1cell lines, in which the expression of mouse Na_x_ fused to the FLAG tag at its amino terminus is inducible, were established as follows. pTRE-FLAG-mNa_x_ was generated by inserting cDNA encoding the FLAG-tagged mouse Na_x_ [15] into a pTRE plasmid (Clontech). Neuro-2a and rat C6 cells were co-transfected with pTRE-FLAG-mNa_x_ together with pcDNA3.1, carrying the neomycin-resistance gene, and selected with 0.5 mg/ml G418. Two clones (C6Mf4 from C6 and N2a-Mf1 from Neuro-2a), which were isolated by limiting dilutions, were used in this study. The induced expression of full-length FLAG-Na_x_ proteins was verified by Western blotting.

### Specific antibodies against mouse Na_x_

The GST fusion protein with the interdomain II-III (amino acid residues 724–933) of mouse Na_x_ was expressed using the expression plasmid pGEX-Na_x_-ID2/3 in *Escherichia coli* strain BL21, and purified by glutathione affinity chromatography. Antisera were prepared using rabbits immunized with the purified protein and Freund’s complete adjuvant (Scrum Inc.). Immunoglobulin fractions were obtained by precipitation with ammonium sulfate at 33% (w/v) saturation. The specific anti-mNa_x_ fraction was prepared by passing through Sepharose (GE Healthcare) conjugated with GST.

### Immunohistochemistry

Mice were anesthetized, and transcardially perfused with a solution containing 137 mM NaCl, 2.7 mM KCl, and 10 mM phosphate buffer, pH 7.3 (PBS), and followed by 10% neutral formalin (Wako Pure Chemical Industries). Dissected brains were post-fixed overnight and embedded in paraffin. After removing paraffin, tissue sections (7-μm thick) were microwaved in 10 mM citrate buffer, pH 6.0 for 15 min, and treated with 3% H_2_O_2_ in 150 mM NaCl, 10 mM Tris-HCl, pH 7.4 (TBS) for 15 min. They were then blocked with a blocking buffer (4% skim milk and 0.1% Tween-20 in TBS), and then incubated with the anti-mNa_x_ antibody. The binding antibodies were detected with the DAKO Envision System (DAKO) or appropriate fluorescent secondary antibodies. The antibodies used are listed in S1 Table.

### Immunocytochemistry

Cells were fixed by layering 5% formaldehyde in PBS containing 20% sucrose at 37°C for 30 min, blocked with the blocking buffer, and then incubated with anti-mNa_x_ and mouse anti-β-tubulin III in the blocking buffer. Bound antibodies were visualized with appropriate fluorescent secondary antibodies. Fluorescence was observed with a wide-field fluorescence microscope (BZ8000, Keyence) or laser scanning confocal microscope (A1R, Nikon). The densitometric analysis of fluorescence intensity was performed as previously described [16]. The antibodies used are listed in S1 Table.

### Reverse transcription polymerase chain reaction (RT-PCR) analysis

Total RNA was isolated from Neuro-2a cells with TRIzol Reagent (Life Technologies). cDNA was synthesized from DNase I-treated total RNA with Superscript III reverse transcriptase (Life Technologies) and subjected to PCR for mouse Na_x_. Mouse glyceraldehyde-3-phosphate dehydrogenase (GAPDH) was used as a control to adjust the amount of mRNA. RT-PCR was performed using primers in the TaqMan Gene Expression assay for Na_x_ (ID#Mm008801952_m1) and GAPDH (ID#Mm99999915_g1) (Applied biosystems).

### Western blotting

Cells (~10^7^ cells) were homogenized in Tris-buffered saline containing 1% Triton X-100 for the Western blot analyses of Neuro-2a cells. After centrifugation at 15,000 g for 15 min, the supernatant was separated by sodium dodecyl sulfate polyacrylamide gel electrophoresis (SDS-PAGE) followed by transfer to a polyvinylidene fluoride (PVDF) membrane (Immobilon-P, Millipore). The blotted membrane was probed with the anti-mNa_x_ antibody as a primary antibody, followed by detection with a corresponding horseradish peroxidase (HRP)-conjugated secondary antibody. Western blot analyses of the pull-down sample using an anti-PSD95 antibody (7E3-1B8, Calbiochem) was performed as described previously [17]. The antibodies used are listed in S1 Table.

### GST pull-down experiment and mass spectrometry

GST-Na_x_ is a GST fusion protein at the C-terminus (amino acid residues 1489–1681) of mouse Na_x_ (GenBank accession no. NM_009135). pGEX-Na_x_ was prepared by subcloning Na_x_ cDNA from pTRE-mNa_x_ [14] into pGEX-6P (GE Healthcare) to express GST-Na_x_. The GST-Na_x_ protein was expressed in the *E*. *coli* strain BL21, and purified by glutathione affinity chromatography as described previously [15].

In pull-down experiments, glutathione Sepharose beads (20 μl) were coated with GST fusion proteins (2 μg), and then incubated overnight at 4°C with synaptosomal lysate (200 μg protein) prepared from the adult rat cerebrum, as described previously [17]. After washing the beads, the bound proteins were solubilized, separated by SDS-PAGE, and stained with Coomassie Brilliant Blue. Specific bands were excised, subjected to in-gel tryptic digestion, and then applied to matrix-assisted laser desorption ionization-time of flight mass spectrometry (MALDI-TOF MS) (Reflex III, Bruker Daltonics). Peptide mass fingerprinting was performed by a Mascot search (http://www.matrixscience.com/) against the NCBI nonredundant protein database.

### Immunoprecipitation experiments

HEK293T cells were transfected with pFLAG-mNa_x_ or pFLAG-mNa_x_-T1679A [15] together with pcDNA-PSD95 [17] using the standard calcium phosphate method. Cells were cultured with DMEM containing 10% FBS under 5% CO_2_ at 37°C for 2 days, and then lysed with lysis buffer (1% Triton X-100 and 150 mM NaCl in 10 mM Tris-HCl, pH 7.4) containing protease inhibitors (Complete Protease Inhibitor Cocktail, Roche Applied Science). Cell extracts were incubated with an anti-FLAG M2 antibody, and the immunocomplexes were precipitated using protein G-Sepharose. After washing the beads, the bound proteins were separated by SDS-PAGE, and followed by Western blotting with anti-PSD95 and anti-mNa_x_ antibodies as described above. The antibodies used are listed in S1 Table.

### RNA interference

Predesigned small interfering RNA (siRNA) against mouse PSD95 (SASI_Mm02_00304274) and control siRNA (MISSION siRNA Universal Negative Control, SIC-001) were purchased from Sigma-Aldrich. siRNAs were transfected into cells using Lipofectamine 2000 (Life technologies), and these cells were then used for experiments after a 36-h culture.

### Na^+^ imaging

Intracellular Na^+^ imaging with sodium-binding benzofuran isophthalate acetoxymethyl ester (SBFI/AM; Molecular Probes) was performed as described previously [12]. The 145 mM Na^+^-recording solution (isotonic solution) contained (in mM): 135 NaCl, 5 KCl, 2.5 CaCl_2_, 1 MgCl_2_, 20 HEPES, and 10 NaOH, titrated to pH 7.3 with HCl. NaCl was added to or removed from the recording solution to achieve the appropriate [Na^+^].

### Patch-clamp experiments

Patch-clamp experiments were performed as previously described with minor modifications [18]. The basal recording solution contained (in mM): 140 NaCl, 5 KCl, 2.5 CaCl_2_, 1 MgCl_2_, 5 HEPES, and 20 glucose. NaCl was added to or removed from the recording solution to achieve the appropriate [Na^+^]. In the experiments to test the ion selectivity of Na_x_ channel, NaCl in the recording solution was replaced with an equivalent amount of the test salt. The pipette solution contained (in mM): 120 K-gluconate, 20 TEA-Cl, 2 MgCl_2_, 2 Na_2_ATP, 1 EGTA, and 10 HEPES (pH 7.3). Cells were voltage clamped at—60 mV during the recordings. In order to detect Na^+^-dependent currents, extracellular solutions were changed using the fast application method with a double-barreled application pipette [19]. The pipette was operated by a piezoelectric device (PZ-150M, Burleigh Instruments). [Na^+^]_o_ at the half-maximal response (C_1/2_) of the [Na^+^]_o_-dependence curve was determined by curve fitting using the equation: I = I_Max_/{1 + exp[(C_1/2_—C)/a]}, where I is the current density and C is [Na^+^]_o_. The value, I_Max_ = 1.0 was used for the calculation. The half maximal ‘C_1/2_’ and value ‘a’ were determined by curve fitting.

### Statistical Analysis

Data were tested for significance with Kyplot software (Kyens). p < 0.01 was considered significant. Data are shown as the mean ± SE.

## Results

### Expression of Na_x_ in neurons

**Fig 1:**
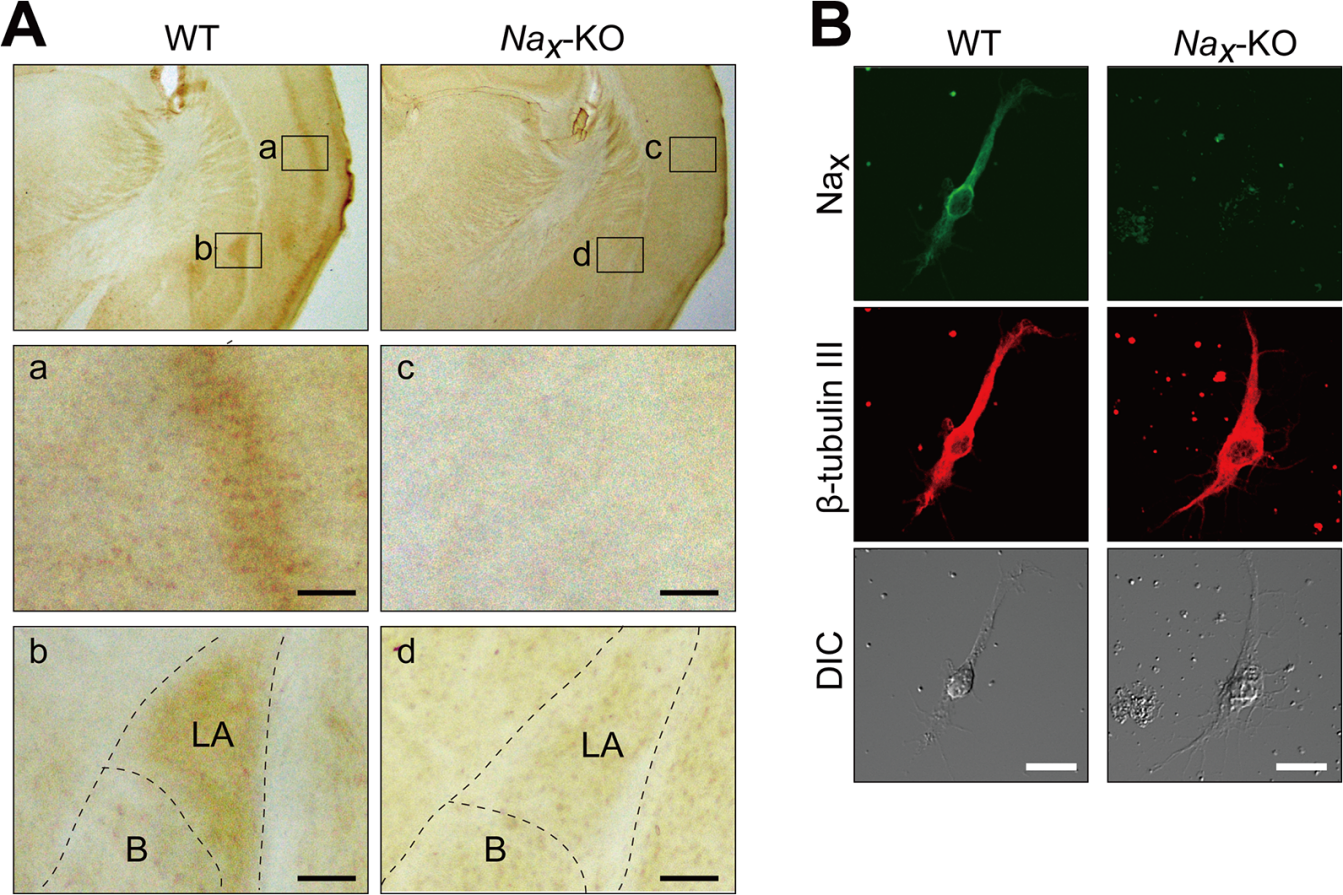
Lateral amygdala neurons express Na_x_ channels. (**A**) Immunohistochemical staining of the coronal sections of adult wild-type (WT) and *Na*_***x***_-knockout (*Na*_***x***_-KO) mice with an anti-mNa_**x**_ antibody. The lower panels are magnified views of the square areas inside the upper panel. Immunohistochemical brown staining was observed in the cortex (a) and lateral amygdala (b) in WT mice, but not in *Na*_***x***_-KO mice (c and d). LA, Lateral amygdala; B, Basal amygdala. Scale bars, 50 μm. (**B**) Double immunofluorescence staining of primary cultured cells obtained from the lateral amygdala of WT and *Na*_***x***_-KO mice with anti-mNa_**x**_ (green) and anti-β-tubulin III (red, a neuronal marker) antibodies. DIC, differential interference contrast image. Scale bars, 20 μm.

In order to verify the expression of Na_x_ in the mouse cortex and amygdala [9] by immunohistochemistry, we newly generated antibodies using the interdomain II-III of mouse Na_x_ (anti-mNa_x_, see the Materials and Methods for details). Using this anti-mNa_x_, we successfully detected the expression of Na_x_ in the cortex and amygdala (Fig 1A, WT), which was previously identified by *lacZ* expression in *Na*_*x*_-KO mice [9]. In the amygdala, the distribution of Na_x_ was restricted to the lateral part (Fig 1A and 1B). The signals in these loci were absent in *Na*_*x*_-KO mice, indicating the specificity of the signals (Fig 1A and 1D; *Na*_*x*_-KO). Immunocytochemistry using a primary culture of the mouse lateral amygdala further showed that Na_x_ was expressed in neurons because they co-localized with the neuronal marker, β-tubulin III (Fig 1B).

### Establishment of Na_x_-expressing neuronal cells

**Fig 2:**
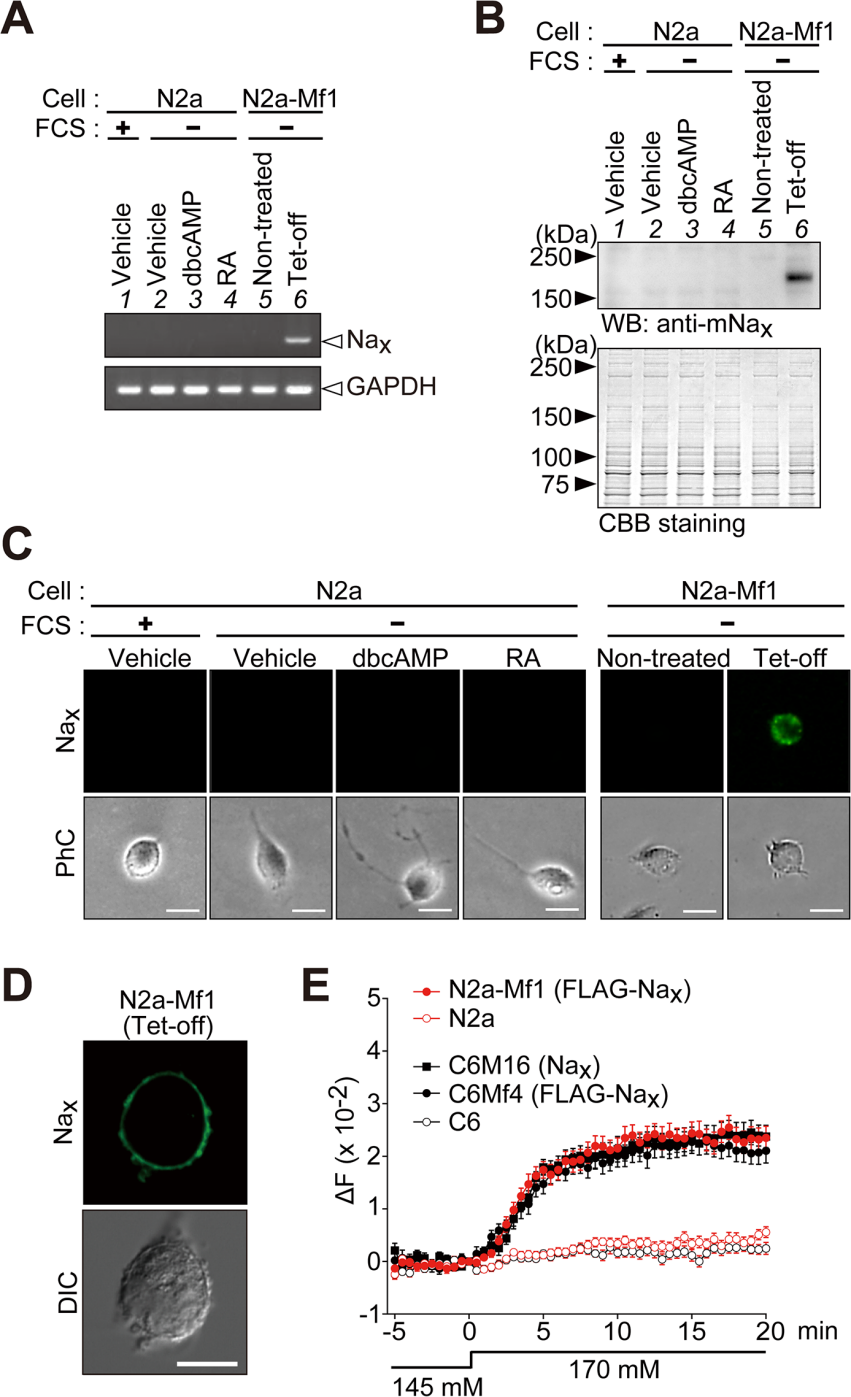
Establishment of mouse neuroblastoma Neuro-2a cells that inducibly express Na_x_. (**A**) A RT-PCR analysis of the expression of Na_**x**_ (upper) and glyceraldehyde 3-phosphate dehydrogenase (GAPDH, lower). Lane 1, parental Neuro-2a (N2a) cells cultured in DMEM containing 10% FCS; lanes 2–4, Neuro-2a cells cultured in serum-free medium with vehicle (lane 2), 1 mM dbcAMP (lane 3), or 20 μM retinoic acid (RA) (lane 4); lanes 5 and 6, a stable transfectant of Neuro-2a cells with pTRE-FLAG-mNa_**x**_ (N2a-Mf1) maintained under serum-free conditions (lane 5) and N2a-Mf1 cells infected with the Tet-off adenovirus for the expression of FLAG-mNa_**x**_ (lane 6). The parental N2a cells were Na_**x**_-negative. GAPDH was amplified from the same cDNA preparation as Na_**x**_. (**B**) Western blotting with anti-mNa_**x**_. Cells were cultured as in **A**. The lower panel shows Coomassie Brilliant Blue (CBB) staining to verify the amount of protein applied. (**C** and **D**) Anti-Na_**x**_ immunocytochemistry of cells cultured as in **A** using a wide-field fluorescence microscope (**C**) and confocal laser scanning microscope (**D**). PhC, phase contrast; DIC, differential interference contrast image. Scale bars, 20 μm for (**C**) and 10 μm for (**D**). (**E**) Na^+^ imaging to confirm the functional expression of FLAG-tagged Na_**x**_ in N2a-Mf1 cells. C6M16 cells expressing non-tagged mouse Na_**x**_ and C6Mf4 cells expressing FLAG-tagged Na_**x**_ were analyzed to determine whether the FLAG tag affected the gating of Na_**x**_ channels. C6M16, C6Mf4, and N2a-Mf1 cells showed similar [Na^+^]_**o**_-sensitive responses. Their parental N2a and C6 cells, which were Na_**x**_-negative, did not show [Na^+^]_**o**_-sensitive responses. The ordinate shows the change observed in the fluorescence ratio (ΔF, 340/380 nm). The fluorescence ratio at 0 min was set as the zero point on the ordinate. The extracellular perfusion solution was changed from the 145 mM Na^+^ solution to the 170 mM Na^+^ solution at 0 min. Data represent the mean ± SE (n = 25 for each). Uncropped images of gels and blots are shown in S3 Fig.

To characterize Na_x_ channel properties in neuronal cells, we attempted to establish stable cells expressing Na_x_ using neuronal cell lines. We examined whether the mouse neuroblastoma Neuro-2a cell line was devoid of the endogenous expression of Na_x_ by RT-PCR (Fig 2A, lane 1). The expression of Na_x_ was not detected in Neuro-2a cells even when they were cultured in serum-depleted (differentiation-inducing) medium or in medium containing dbcAMP or retinoic acid (Fig 2A; lanes 2–4).

We first established C6Mf4 cells, a C6 cell line expressing FLAG-tagged mouse Na_x_, and compared the [Na^+^]-sensitive responses of the FLAG-tagged Na_x_ in C6Mf4 to those of non-tagged Na_x_ in C6M16 cells [14] using Na^+^-imaging experiments. The expression of Na_x_ channels was inducible in these cells under the control of TRE (see the Materials and Methods, [14]). When [Na^+^]_o_ was increased from 145 mM to 170 mM, C6Mf4 cells expressing FLAG-tagged Na_x_ exhibited increases in intracellular Na^+^ concentrations ([Na^+^]_i_), as did C6M16 (Fig 2E, C6M16 and C6Mf4). The time courses of these cells were similar, indicating that the FLAG tag did not affect the gating of Na_x_ channels.

Because Neuro-2a was found to be available for the host cell as described above, we then attempted to establish a cell line using Neuro-2a cells, which are inducible for the expression of FLAG-tagged Na_x_. When Neuro-2a cells were treated with the Tet-off vector, the expression of Na_x_ was detected by RT-PCR (Fig 2A, lanes 5 and 6). These RT-PCR results were confirmed by a Western blot analysis (Fig 2B) and immunocytochemistry using a wide-field fluorescence microscope (Fig 2C). When we used a confocal microscope to observe the immunostained cells, Na_x_ signals were mainly observed at the plasma membrane, indicating the cell-surface expression of Na_x_ (Fig 2D): Neuro-2a cells endogenously expressed PSD95, which promotes the cell-surface expression of Na_x_ (see below). We named the cell line thus obtained N2a-Mf1.

We examined the function of Na_x_ in N2a-Mf1 using FLAG-tagged Na_x_. When [Na^+^]_o_ was increased from 145 mM to 170 mM, N2a cells expressing FLAG-tagged Na_x_ showed increases in [Na^+^]_i_ (Fig 2E, N2a-Mf1), indicating that Na_x_ opened in a [Na^+^]-dependent manner also in neurons. The [Na^+^]-sensitive responses of these cells were very similar, suggesting that the channel properties of Na_x_ were not affected by the host cell (Fig 2E, compare N2a-Mf1 with C6Mf4).

Their parental Neuro-2a and C6 cells, which were Na_x_-negative, did not show this increase in [Na^+^]_i_. These results indicated that Na_x_ channels expressed in neurons, as well as those in glial cell, were functional and responded to increase in [Na^+^]_o_.

### Na^+^ sensitivity of Na_x_ in neuronal cells is similar to that in glial cells

**Fig 3:**
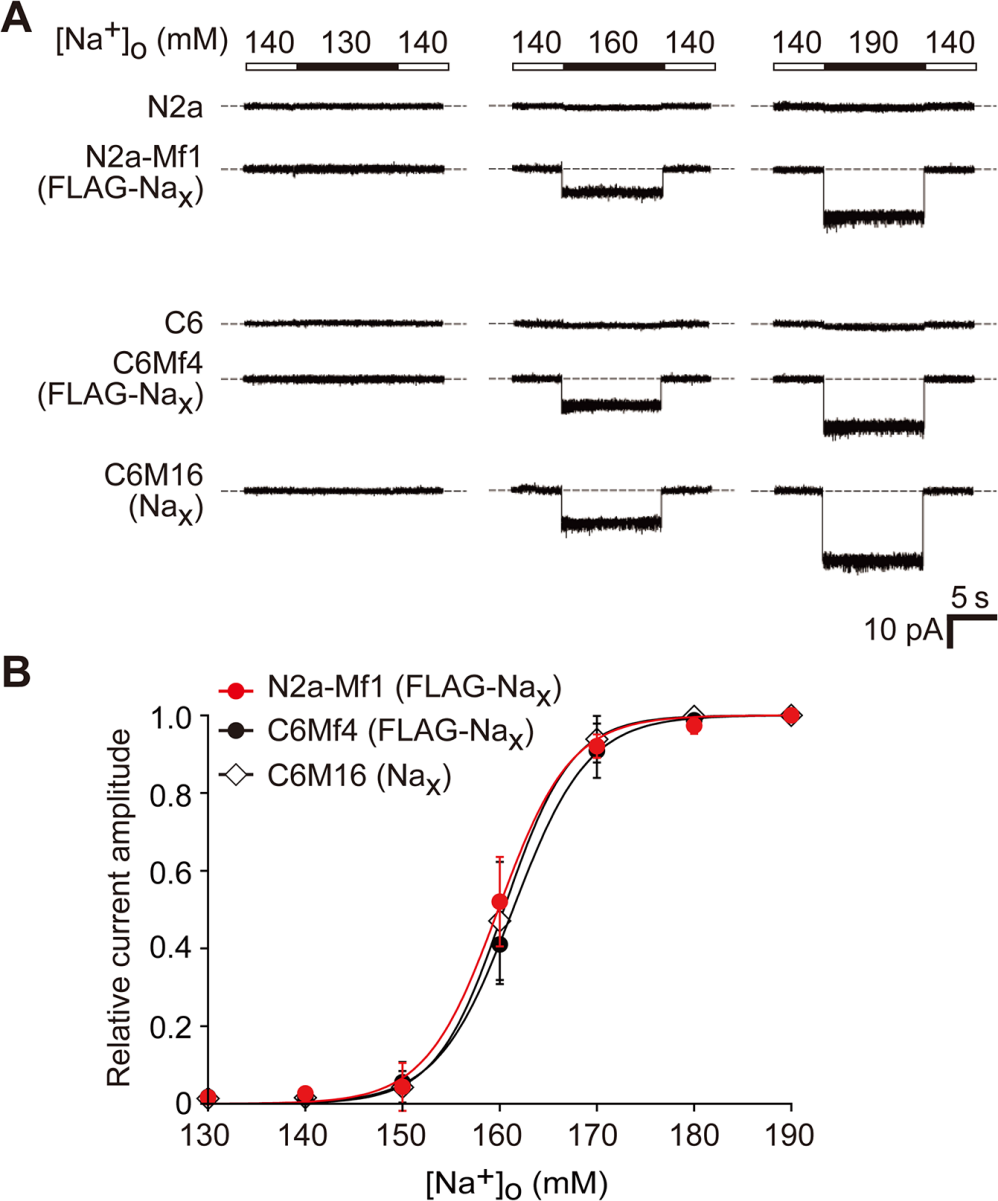
Comparison of Na^+^ sensitivity of Na_x_ expressed in neuronal Neuro-2a and glial C6 cells. (**A**) A comparison of the sodium sensitivity of Na_**x**_ channels between Neuro-2a and C6 transfectants by whole-cell patch-clamp recording. Representative whole-cell current responses by the application of hypotonic (130 mM) or hypertonic (160 and 190 mM) solution of [Na^+^]_**o**_ were shown. (**B**) Relationships between the relative current amplitude and [Na^+^]_**o**_. Each current amplitude was normalized to the amplitude of the current elicited by a solution change to 190 mM [Na^+^]_**o**_. Data represent the mean ± SE.

We measured the current response of Na_x_ expressed in N2a-Mf1 to [Na^+^]_o_ changes using a patch-clamp method with a voltage-clamp configuration. When the “high Na^+^ solution” ([Na^+^]_o_ = 160 and 190 mM) was applied to the cells, inward currents were observed in Na_x_-expressing cells (Fig 3A, N2a-Mf1, middle and right columns). It was not inactivated under the high Na^+^ solution conditions, but disappeared rapidly when [Na^+^]_o_ was returned to the basal level ([Na^+^]_o_ = 140 mM). No inward currents were observed when [Na^+^]_o_ was lowered from the control amount of 140 mM to 130 mM (Fig 3A, N2a-Mf1, left column). These responses were very similar to those observed in C6Mf4 and C6M16 (Fig 3A, C6Mf4 and C6M16), but were not observed in Neuro-2a or C6 cells (Fig 3A N2a and C6). We further examined the relationship between the relative current amplitude and [Na^+^]_o_ (Fig 3B). The response curve of the [Na^+^]_o_ dependency of Na_x_ observed in Na_x_-expressing Neuro-2a cells was similar to that in Na_x_-expressing C6 cells (the half maximums of the curves for N2a-Mf1, C6Mf4, and C6M16 were 159.8, 161.4, and 160.4, respectively).

### Ion selectivity of the cation-sensitive response of Na_x_ channels

**Fig 4:**
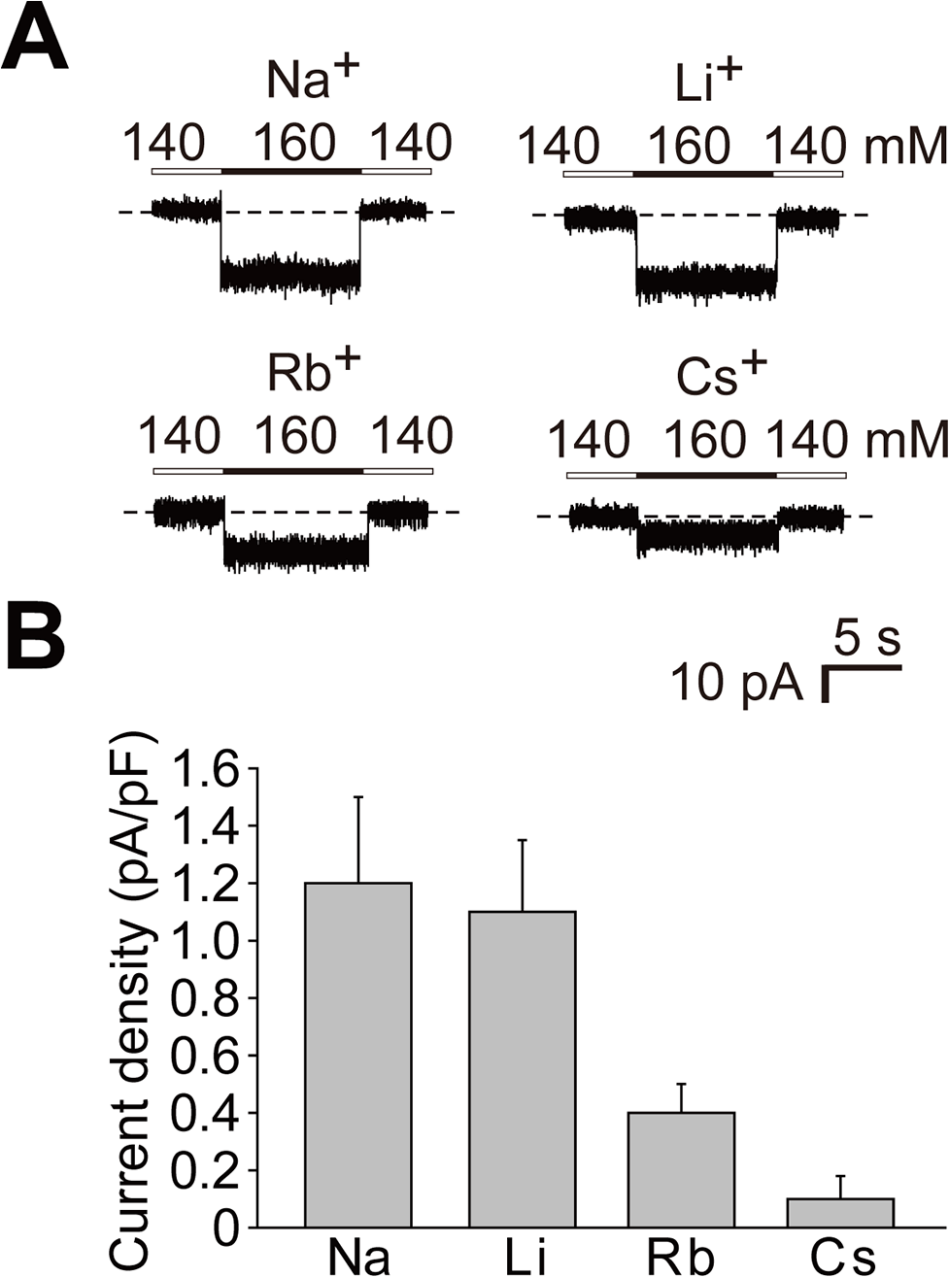
Ion selectivity of the cation-sensitive response of the Na_x_ channel. (**A**) Representative whole-cell current responses in N2a-Mf1 expressing FLAG-Na_**x**_ elicited by the addition of 20 mM of the test cations as chloride salt. (**B**) Summary of whole-cell current responses in **A**. Data represent the mean ± SE. n = 5.

We examined current responses to different monovalent ions using N2a-Mf1 cells expressing Na_x_ channels. When extracellular Na^+^ at 160 mM was completely replaced with lithium ions (Li^+^), rubidium ions (Rb^+^), or cesium ions (Cs^+^), the current density (current amplitude normalized with cell capacitance) decreased in this order (Fig 4). The current amplitude for each ion species remained unchanged when the concentration (160 mM for each cation) was maintained, but immediately disappeared when the extracellular concentration was returned to the basal level (140 mM for each cation). Therefore, these current properties were similar to those for Na^+^.

### Na_x_ interacts with PSD95 through its PDZ-binding motif

**Fig 5:**
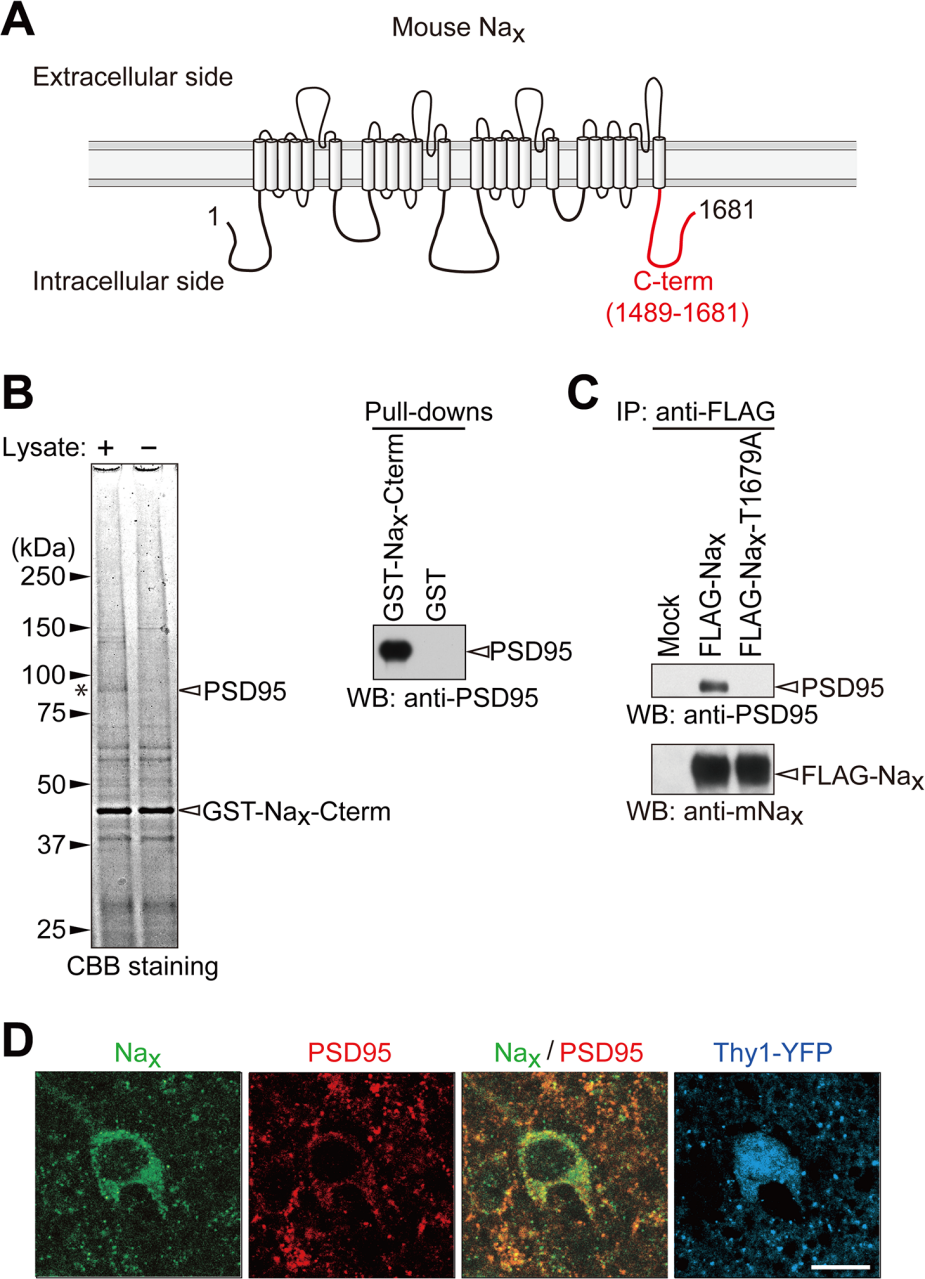
Na_x_ binds to PSD95 via its C-terminal PDZ-domain binding motif. (**A**) Schematic diagram of mouse Na_**x**_. The C-terminal region used for the pull-down experiment is indicated by a red line. Numbers refer to amino acid residues. (**B**) GST pull-down assays. Left, CBB-stained gel of pull-down samples. The asterisk indicates the main band, which was specifically detected in the pull-down samples using GST-Na_**x**_-Cterm. The main band at 95 kDa was identified as PSD95 by mass spectrometry. Right, Western blot analyses of pull-down samples from the synaptosome fraction of the rat cortex using GST-Na_**x**_-Cterm. Western blotting was performed with an anti-PSD95 antibody. (**C**) Binding of full-length Na_**x**_ to PSD95 in HEK293T cells. The expression construct for PSD95 was transfected into HEK293T cells, together with the control vector (Mock), FLAG-tagged wild-type Na_**x**_, or its FLAG-tagged PDZ-binding-motif mutant. The upper box shows the sequence of the PDZ-binding motif of mouse Na_**x**_ and its mutant. The lower panels show immunoprecipitation. The amounts of protein immunoprecipitated with anti-FLAG M2 were analyzed by Western blotting using anti-PSD95 and anti-mNa_**x**_ antibodies. The amounts of protein expressed in the cell extract were shown in S1 Fig. (**D**) Immunohistochemical staining of the lateral amygdala in the Thy1-YFP mouse with anti-mNa_**x**_ (green) and anti-PSD95 (red). The fluorescence signals of YFP are indicated in blue. YFP was expressed in a subset of neurons in the brain of the Thy1-YFP mouse. Scale bars, 10 μm. Uncropped images of blots are shown in S1 and S3 Figs.

Na_x_ on the plasma membrane of glial cells is known to be stabilized by binding to SAP97 through its C-terminus [15]; therefore, we assumed that other PDZ proteins exist in neurons that interact with the C-terminus of Na_x_ and promote the cell-surface expression of Na_x_ in neurons. We performed pull-down experiments using the Na_x_ C-terminal region fused with GST (GST-Na_x_-Cterm, see Fig 5A) from the synaptosomal fraction of the cerebral cortex of adult rats. Several specific bands bound for GST-Na_x_-Cterm were detected in the pulled-down fraction (Fig 5B, left panel). The main band at 95 kDa was identified as PSD95 by mass spectrometry. We confirmed interactions between the C-terminal region of Na_x_ and PSD95 by Western blotting of the pull-downed sample with GST-Na_x_-Cterm (Fig 5B, right panel). We also identified an interaction between the full-length Na_x_ and full-length PSD95 by immunoprecipitation using cell extracts from HEK293T cells in which the expression vectors of FLAG-tagged Na_x_ and PSD95 were co-transfected (Fig 5C and S1 Fig). PSD95 was not immunoprecipitated with mouse Na_x_ with a mutation at the PDZ-binding motif (FLAG-Na_x_-T1679A) (Fig 5C). This result indicated that PSD95 bound to Na_x_ through the C-terminal PDZ-binding motif of Na_x_, as was the case for SAP97 [15]. Double immunostaining of sections of the mouse brain showed that Na_x_ and PSD95 were co-expressed at the cellular level in Thy1-positive neurons in the amygdala (Fig 5D).

### PSD95 promotes the stability of Na_x_ at the plasma membrane

**Fig 6:**
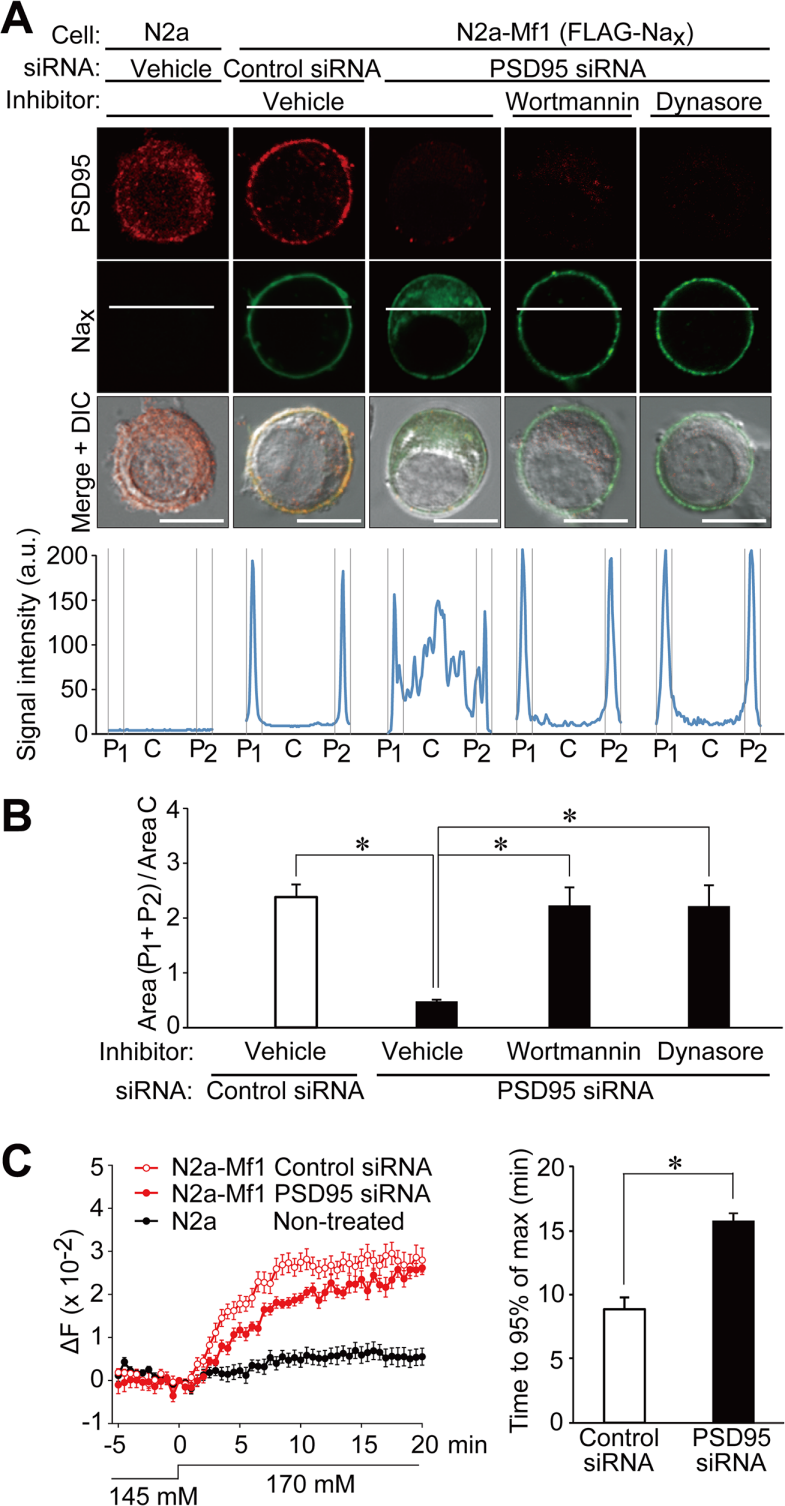
PSD95 promotes the stability of Na_x_ channels at the plasma membrane in neuronal cells. (**A**) Subcellular distribution of Na_**x**_ in non-treated Neuro-2a (N2a) cells, or N2a-Mf1 cells transfected with control or PSD95 siRNA. Upper panels: Immunostaining with anti-PSD95 and anti-mNa_**x**_ antibodies. In order to inhibit endocytosis, cells expressing Na_**x**_ were treated with 100 nM wortmannin or 200 μM dynasore for 6 h. These cells were then fixed, permeabilized, and stained with anti-mNa_**x**_. Scale bars, 10 μm. Lower graphs: Fluorescence intensity profiles along the white lines in the upper panels. The profile was divided into three parts (P1: C: P2 = 15: 70: 15 in length). a.u., arbitrary unit. (**B**) The relative fluorescence intensity of the membrane region to the central region in **A**. Data represent the mean ± SE (n = 8 for each); *p < 0.01, ANOVA followed by Scheffe’s test. (**C**) Reduced Na^+^ influx in N2a-Mf1 cells in the absence of PSD95. Left panel: Na^+^ imaging of N2a-Mf1 cells transfected with PSD95 or control siRNA, or non-treated N2a cells. Data represent the mean ± SE (n = 21 for each). Right panel: Summary of the time taken to reach 95% of the plateau level. Data represent the mean ± SE (n = 21 for each); *p < 0.01, two-tailed t test.

In parental Neuro-2a cells, endogenous PSD95 was detected in the intracellular region (Fig 6A, N2a; see also S2 Fig, Non-treated). We examined the subcellular distribution of endogenous PSD95 and heterologously expressed Na_x_. In N2a-Mf1 cells that expressed Na_x_, PSD95 was clearly observed at the plasma membrane in addition to the cytoplasm, whereas Na_x_ was localized to the plasma membrane (Fig 6A and 6B, N2a-Mf1, Control siRNA).

We previously demonstrated that SAP97 contributed to the stabilization of Na_x_ at the plasma membrane in glial cells. To determine whether the surface expression of Na_x_ channels was affected by endogenous PSD95, we transfected siRNA to knockdown PSD95 in N2a-Mf1 cells. Along with a decrease in the expression of PSD95 (S2 Fig), the surface expression of wild-type Na_x_ was found to be markedly decreased (Fig 6A and 6B, N2a-Mf1, PSD95 siRNA, Vehicle). An incubation with wortmannin, an inhibitor of endocytosis [20], or dynasore, an inhibitor for dynamin-dependent endocytosis [21], markedly ameliorated the surface expression of Na_x_ (Fig 6A and 6B, Wortmannin and Dynasore). These results indicated that binding to PSD95 promoted the stabilization of Na_x_ at the plasma membrane.

We next investigated whether the reduction in cell surface Na_x_ by the treatment with PSD95 siRNA resulted in the suppression of Na^+^ influx by Na_x_. When [Na^+^]_o_ was increased from 145 mM to 170 mM, N2a-Mf1 cells showed increases in [Na^+^]_i_, and this level eventually reached an equilibrium point between Na^+^ influx by Na_x_ and Na^+^ export by Na^+^/K^+^-ATPase (Fig 6C, left panel). However, when the expression of PSD95 was knocked down with siRNA, the time taken to reach the plateau of [Na^+^]_i_ was markedly prolonged (Fig 6C, right panel).

## Discussion

In the present study, we confirmed that Na_x_ was expressed in the neurons of the lateral amygdala. Functional analyses of Na_x_ exogenously expressed in neuronal cells revealed that the [Na^+^]-sensitivity of Na_x_ was similar to that expressed in glial cells. Furthermore, we demonstrated that Na_x_ bound to PSD95 through its PDZ-binding motif at the C-terminus in neurons. The interaction between Na_x_ and PSD95 was crucial for the surface expression of Na_x_.

As showen in Fig 2, the expression of Na_x_ was not detected in Neuro-2a cells not only by RT-PCR (Fig 2A), but also by immunocytochemistry using our anti-mouse Na_x_ antibody (Fig 2B and 2C), irrespective of their differentiation state. [Na^+^]-sensitive responses were not observed in Neuro-2a cells by our Na^+^-imaging and electrophysiological experiments (Figs 2E and 3A). Na_x_ proteins and [Na^+^]-sensitive responses only appeared when exogenous Na_x_ was expressed (Figs 2 and 3). These results clearly indicated that Neuro-2a cells did not express Na_x_ endogenously.

However, a very recent study reported that the immunocytochemical signals of Na_x_ were detected in Neuro-2a cells [22]. This group postulated that Na_x_ was expressed in neurons in the rat median preoptic nucleus (MnPO) using their antibodies [23]. We examined the expression of Na_x_ by immunohistochemistry using our antibodies to rat Na_x_ and mouse Na_x_ [12 and the present study, respectively]: The specificities of our antibodies were confirmed using tissues and tissue lysates from *Na*_*x*_-KO mice. We did not detect any Na_x_ signals in the MnPO in the rat or mouse brain (S4 Fig), as we have previously discussed [24]. This result is consistent with our previous findings in which *lacZ* signals were negative in the MnPO in *Na*_*x*_-KO mice [9]. Furthermore, they claimed that the Na^+^ leak currents observed in rat MnPO neurons have [Na^+^]-independent conductance [25]. However, we previously demonstrated that Na_x_ had a [Na^+^]_o_-dependent gating property [6, 12]. Collectively, these results indicated that the signals and currents that they described were not derived from Na_x_.

We herein showed that the cation selectivity sequence of Na_x_ was Na^+^ ≈ Li^+^ > Rb^+^ > Cs^+^ (Fig 4). This sequence was similar to those of voltage-gated sodium channels (Na_v_) in myelinated nerves in a previous study [26]. Na_x_ passed certain amounts of Rb^+^ and Cs^+^ (Fig 4B), while the permeability of Na_v_ for Rb^+^ and Cs^+^ was nearly negligible [26]. An ion selectivity filter has been postulated to exist on the extracellular side of the pore of the sodium channel α-subunit: an outer ring with the amino acid sequence EEMD and inner ring with DEKA [27, 28]. These two rings were conserved in all Na_v_ (Na_v_ 1.1–1.9). In contrast, those in Na_x_ were EEID and DENS, respectively, suggesting that the relatively larger permeability to Rb^+^ and Cs^+^ in Na_x_ may be caused by these differences. The best way to estimate the ion selectivity of channel permeability is to determine the permeability ratios for each ion. Measurements of precise reversal potentials for each condition are required to calculate permeability ratios [28]; however, we could not measure precise reversal potentials because the currents were very small. Electrophysiological analyses of purified Na_x_ in a planar phospholipid bilayer are needed to further characterize Na_x_.

Taken together with our previous findings [15], PSD95 and SAP97 both contributed to the surface expression of Na_x_ in neurons and glial cells, respectively. Both PSD95 and SAP97 are members of the membrane-associated guanylate kinase (MAGUK) family, which form a scaffold for the clustering of receptors, ion channels, and associated signaling proteins [29]. As shown in Fig 5D, Na_x_ in the lateral amygdala co-localized with PSD95 clusters, which appeared to exist along dendrites, suggesting that PSD95 played a role in the stabilization of Na_x_ at synapses. On the other hand, Na_x_ in the SFO was localized to perineuronal lamellate processes that extended from glial cells (ependymal cells and astrocytes) [10], suggesting that SAP97 contributed to this localization in glial cells. SAP97 was reported to be expressed at the postsynapses of GABAergic interneurons in the lateral amygdala [30]. Therefore, SAP97 may also be expressed in Na_x_-positive neurons and play a role in the stabilization of Na_x_ at the plasma membrane not only in glial cells, but also in neurons.

Several ion channels have been shown to interact with PSD95 via their C-terminal PDZ-binding motifs: Voltage-gated K channels (Kv1.4, Kv1.5, and Kv4.2), the inward rectifier K channels (Kir channels; Kir2.1, Kir2.3, and Kir5.1), the Na^+^-sensitive K channel (Slo2), the acid-sensing ion channel (ASIC3), and ligand gated glutamate receptor NMDA receptor (NR2) [31–39]. Together with these channel proteins, Na_x_ channels may exist in the postsynaptic density of excitatory synapses in the lateral amygdala and be functionally coupled to these channels through its ion transport. However, it is unlikely that [Na^+^]_o_ around synapses increased in the amygdala under normal conditions. A certain level of endothelins (ETs) has been shown to activate Na_x_ under physiological [Na^+^]_o_ conditions [18, 40]. The opening of Na_x_ channels by ET signaling may depolarize the postsynaptic membrane in neurons through the influx of Na^+^. The physiological roles of Na_x_ in brain neurons including the amygdala will be the subject of future investigations.

## Supporting Information

S1 Fig Supplemental data related to Fig 5C. (**A**) Western blotting of the total cell extracts used in the immunoprecipitation with anti-mNa_x_ (left) and anti-PSD95 (right) antibodies. (**B**) The original blot images of the Western blotting of the immunoprecipitates with anti-mNa_x_ (left) and anti-PSD95 (right) antibodies presented in Fig 5C. Red squares indicate the areas used in the main figures.
(TIF)

S2 Fig Depletion of PSD95 in N2a-Mf1 cells by siRNA. Reduction of PSD95 by PSD95 small interfering RNA (siRNA) in N2a-Mf1 cells was verified by Western blotting with anti-PSD95 antibody (left). The right panel shows CBB staining to verify the amount of protein applied. The expression of PSD95 was reduced in N2a-Mf1 cells transfected with PSD95 siRNA but not with control siRNA.
(TIF)

S3 Fig Original images presented in Fig 2A and 2B, and Fig 5B. (**A**) Original gel images presented in Fig 2A. (**B**) Original blot image presented in Fig 2B. (**C**) Original blot image presented in Fig 5B. Red squares indicate the areas used in the Fig 5B.
(TIF)

S4 Fig Immunohistochemical staining of rat and mouse brains with anti-Na_x_ antibodies. Immunohistochemical staining of the coronal sections of rat and mouse brains, containing the median preoptic nucleus (MnPO) (**A**) and median eminence (**B**) with anti-rat Na_x_ [12] and anti-mNa_x_ antibodies. Immunohistochemical staining was performed as described in S5 File. Neither rat nor mouse MnPO was negative for Na_x_ (**A**). On the other hand, the median eminence was clearly stained with both antibodies (**B**). AC, anterior commissure. Scale bars, 200 μm.
(TIF)

S1 File Supporting method for S4 Fig. (DOCX)

S1 Table Antibodies used for this study. (XLSX)
