# Correction: Channel Properties of Na_x_ Expressed in Neurons

**DOI:** 10.1371/journal.pone.0130107

**Published:** 2015-06-04

**Authors:** The PLOS ONE Staff

S4 Fig is incorrect. Please view the correct S4 Fig below. The publisher apologizes for the error.

## Supporting Information

S4 Fig Immunohistochemical staining of rat and mouse brains with anti-Na_x_ antibodies. Immunohistochemical staining of the coronal sections of rat and mouse brains, containing the median preoptic nucleus (MnPO) (A) and median eminence (B) with anti-rat Na_x_ [12] and anti-mNa_x_ antibodies. Immunohistochemical staining was performed as described in S5 File. Neither rat nor mouse MnPO was negative for Na_x_ (A). On the other hand, the median eminence was clearly stained with both antibodies (B). AC, anterior commissure. Scale bars, 200 μm.
(TIF)

## References

[1] Matsumoto M, Hiyama TY, Kuboyama K, Suzuki R, Fujikawa A, Noda M (2015) Channel Properties of Na_x_ Expressed in Neurons. PLoS ONE 10(5): e0126109 doi:10.1371/journal.pone.0126109 25961826 10.1371/journal.pone.0126109 PMC4427406

