# Supplementary figures and images for: Channel Properties of Nax Expressed in Neurons
Source: PLoS One. 2015 May 11;10(5):e0126109. doi: 10.1371/journal.pone.0126109 (PMC4427406; doi:10.1371/journal.pone.0126109)

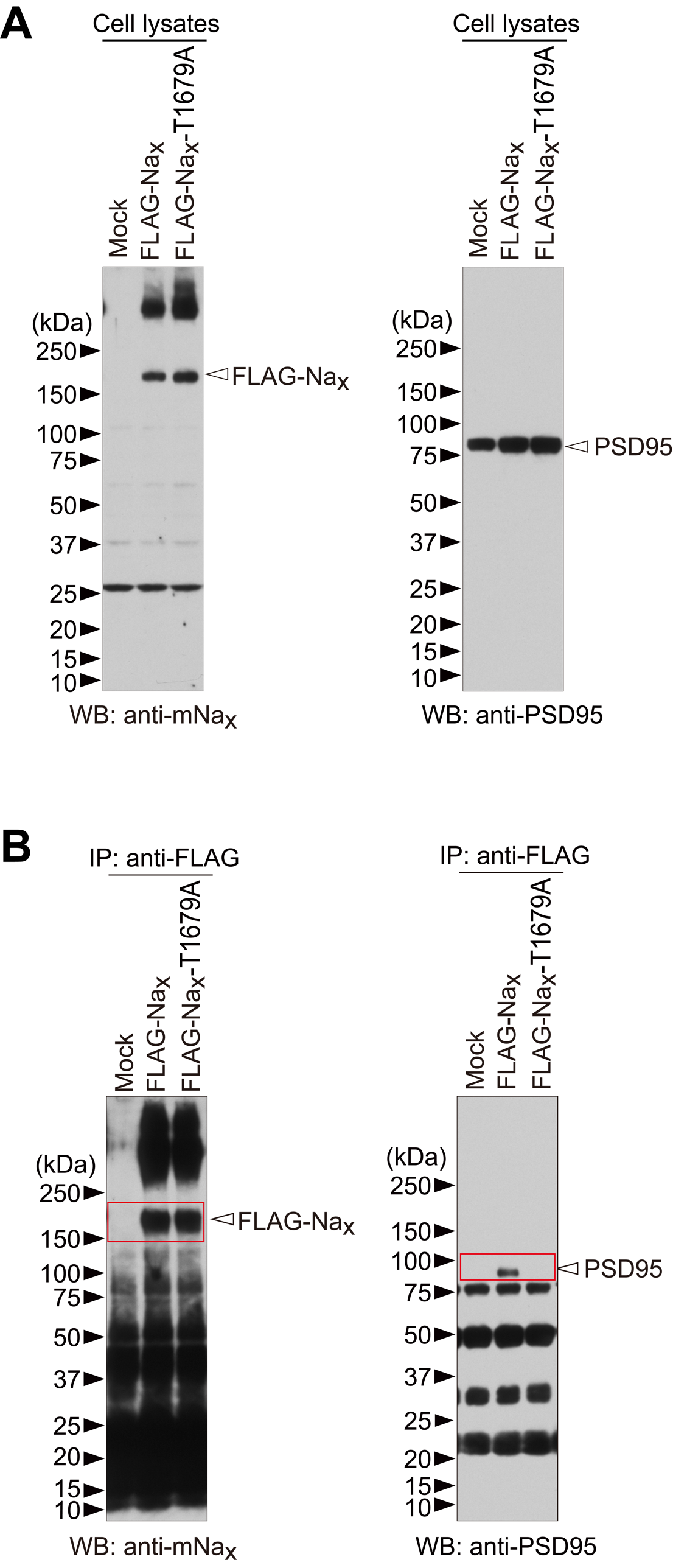

Supplement: S1 Fig — (A) Western blotting of the total cell extracts used in the immunoprecipitation with anti-mNax (left) and anti-PSD95 (right) antibodies. (B) The original blot images of the Western blotting of the immunoprecipitates with anti-mNax (left) and anti-PSD95 (right) antibodies presented in Fig 5C. Red squares indicate the areas used in the main figures. (TIF) [file pone.0126109.s001.tif]

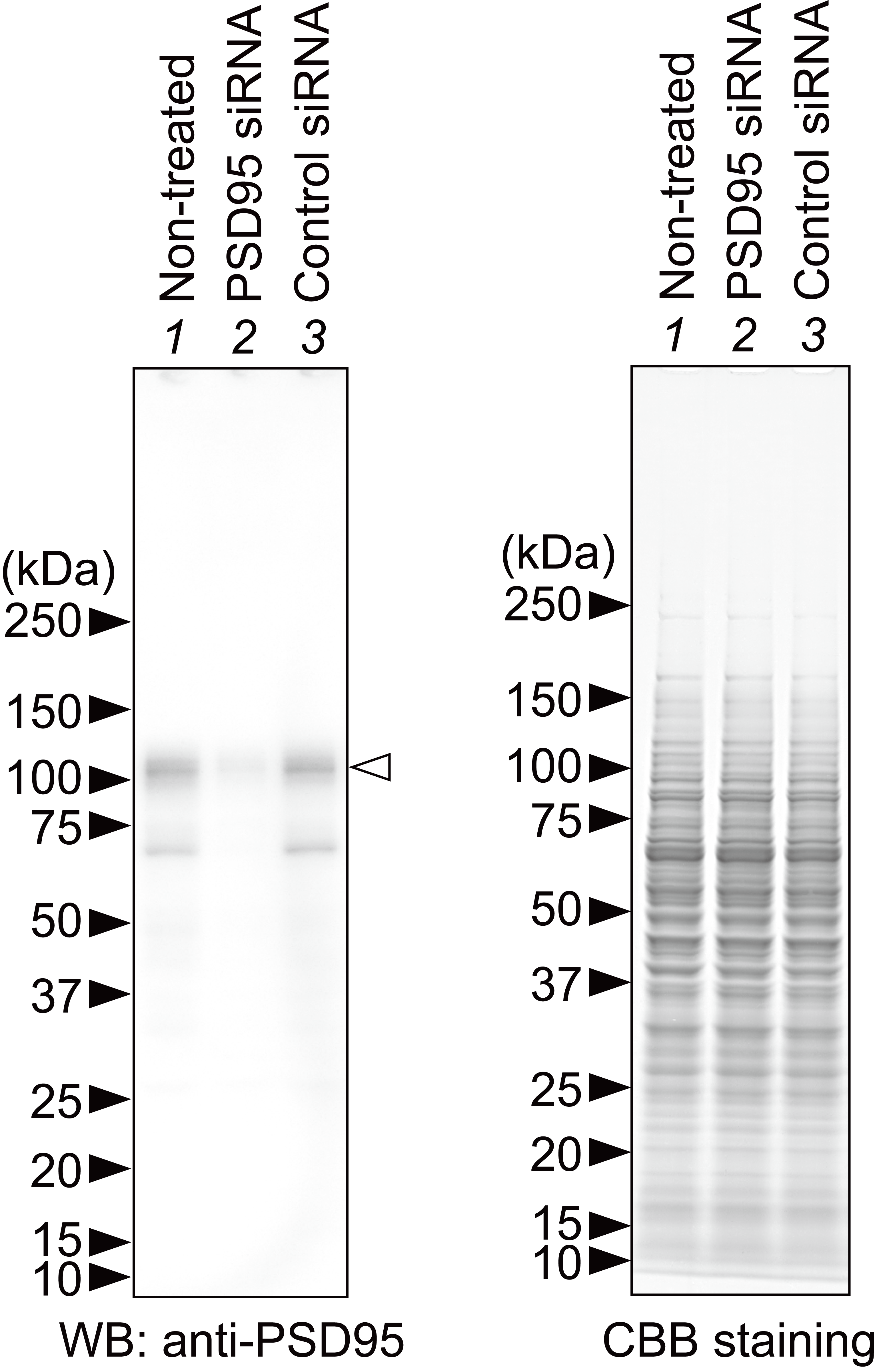

Supplement: S2 Fig — Reduction of PSD95 by PSD95 small interfering RNA (siRNA) in N2a-Mf1 cells was verified by Western blotting with anti-PSD95 antibody (left). The right panel shows CBB staining to verify the amount of protein applied. The expression of PSD95 was reduced in N2a-Mf1 cells transfected with PSD95 siRNA but not with control siRNA. (TIF) [file pone.0126109.s002.tif]

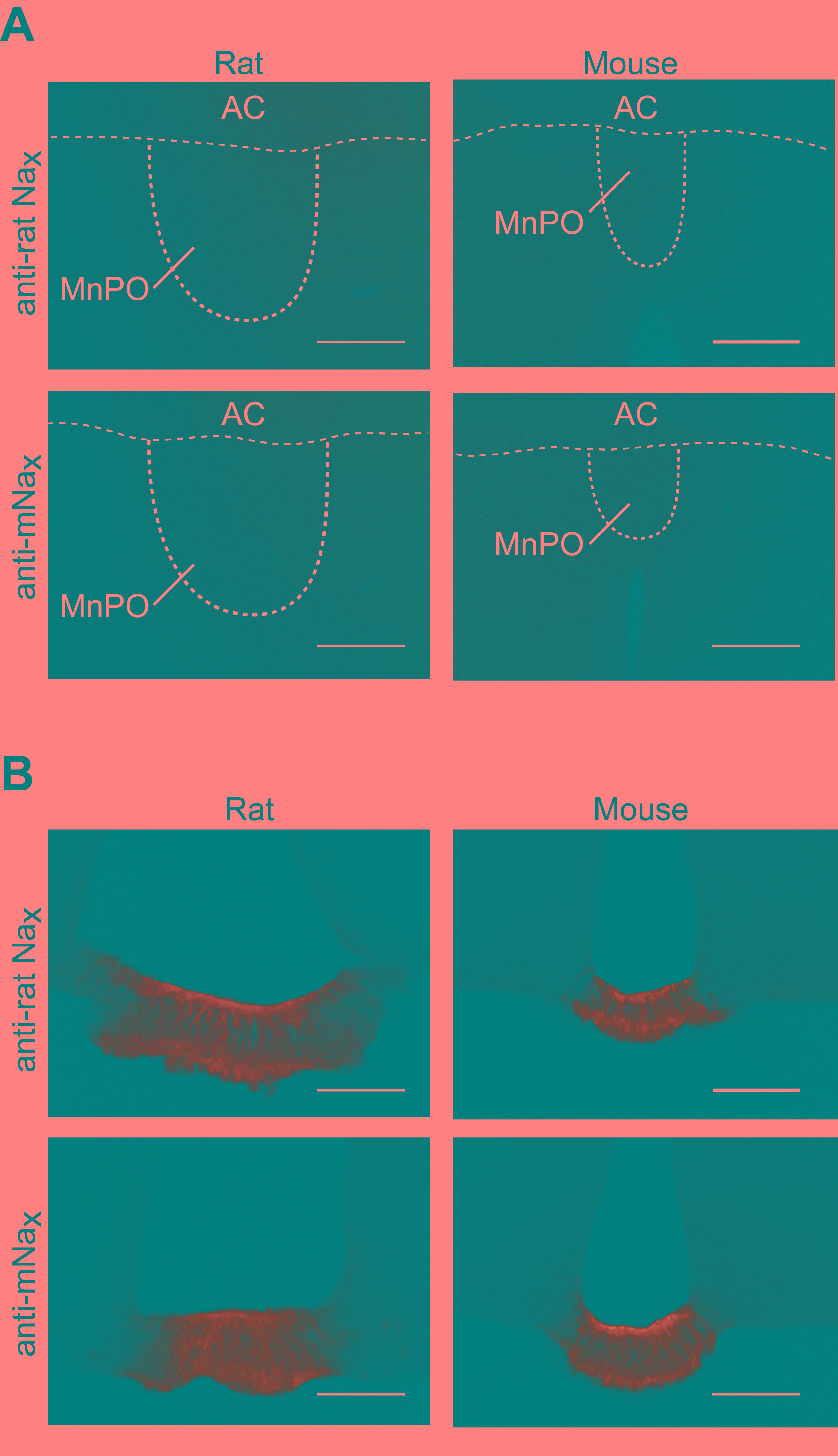

Supplement: S4 Fig — Immunohistochemical staining of the coronal sections of rat and mouse brains, containing the median preoptic nucleus (MnPO) (A) and median eminence (B) with anti-rat Nax [12] and anti-mNax antibodies. Immunohistochemical staining was performed as described in S5 File. Neither rat nor mouse MnPO was negative for Nax (A). On the other hand, the median eminence was clearly stained with both antibodies (B). AC, anterior commissure. Scale bars, 200 μm. (TIF) [file pone.0126109.s004.tif]
