# Supplementary material for: Channel Properties of Nax Expressed in Neurons
Source: PLoS One. 2015 May 11;10(5):e0126109. doi: 10.1371/journal.pone.0126109 (PMC4427406; doi:10.1371/journal.pone.0126109)
Supplement: S1 File — (DOCX) [file pone.0126109.s005.docx]

**Matsumoto et al., S1 File.**

**Supporting method for S4 Fig.**

**Immunofluorescence staining**

Rats and mice were anaesthetized, transcardially perfused with PBS, and followed by 10% neutral formalin. Brains were dissected and post-fixed overnight. After several washes with PBS, the brain samples were cut on a vibratome (VT1000S, Leica) to obtain coronal sections (50 μm). The sections were blocked with a blocking buffer (5% normal goat serum and 0.1% Triton X-100 in PBS), and then incubated with anti-rat Na_x_ or anti-mNax antibodies. The binding antibodies were detected with appropriate secondary antibodies with fluorescence. The antibodies used are listed in S1 Table.
