# Supplementary figures and images for: Correction: Channel Properties of Nax Expressed in Neurons
Source: PLoS One. 2015 Jun 4;10(6):e0130107. doi: 10.1371/journal.pone.0130107 (PMC4456422; doi:10.1371/journal.pone.0130107)

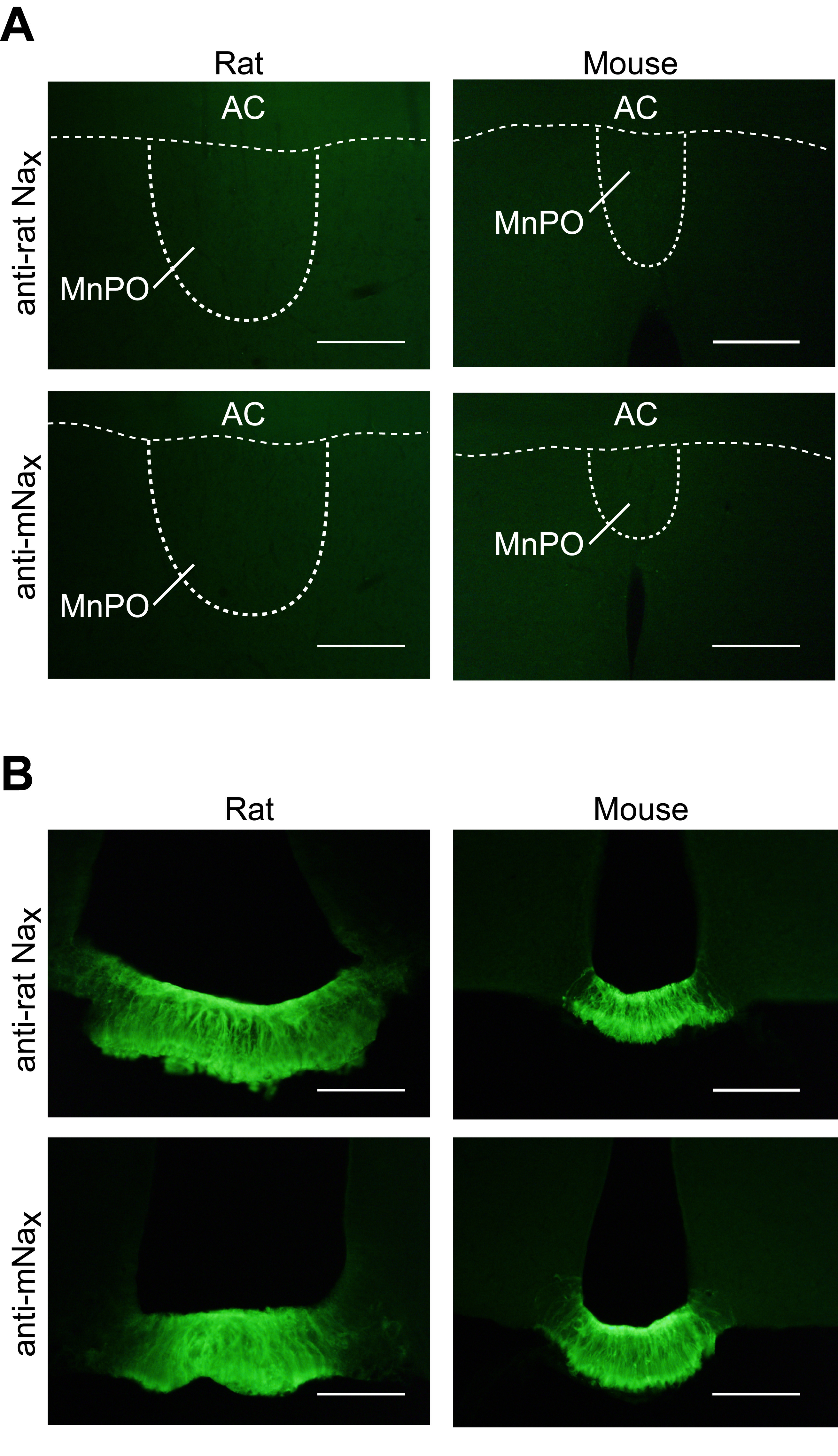

Supplement: S4 Fig — Immunohistochemical staining of the coronal sections of rat and mouse brains, containing the median preoptic nucleus (MnPO) (A) and median eminence (B) with anti-rat Nax [12] and anti-mNax antibodies. Immunohistochemical staining was performed as described in S5 File. Neither rat nor mouse MnPO was negative for Nax (A). On the other hand, the median eminence was clearly stained with both antibodies (B). AC, anterior commissure. Scale bars, 200 μm. (TIF) [file pone.0130107.s001.tif]
